# Geospatial variations in socioeconomic conditions and health outcomes in COVID-19 era: insights from South Africa (2020–2022)

**DOI:** 10.1007/s10708-023-10851-4

**Published:** 2023-03-07

**Authors:** Handan Wand, Cassandra Vujovich-Dunn, Kate Derrick, Jayajothi Moodley, Tarylee Reddy, Sarita Naidoo

**Affiliations:** 1 Biostatistics and Databases Program ,Kirby Institute, University of New South Wales, Level 6, Wallace Wurth Building, Kensington, NSW 2052 Australia; 2 Emergency Department, Royal Prince Alfred Hospital, Sydney, Australia; 3 The Aurum Institute, Johannesburg, South Africa; 4 Biostatistics Unit, South African Medical Research Council, Durban, Kwazulu-Natal South Africa; 5 Numolux Group, Pretoria, South Africa

**Keywords:** Geoadditive models, Food insecurity, Depressive symptoms, South Africa, COVID-19

## Abstract

South Africa also has the highest burden of coronavirus disease 2019 (COVID-19) related comorbidities in Africa. We aimed to quantify the temporal and geospatial changes in unemployment, food insecurity, and their combined impact on depressive symptoms among South Africans who participated into several rounds of national surveys. We estimated the population-attributable risk percent ($PAR\%$) for the combinations of the risk factors after accounting for their correlation structure in multifactorial setting. Our study provided compelling evidence for immediate and severe effect of the pandemic where 60% of South Africans reported household food insecurity or household hunger, shortly after the pandemic emerged in 2020. Despite the grants provided by the government, these factors were also identified as the most influential risk factors (adjusted odds ratios (aORs) ranged from 2.06 to 3.10, *p* < 0.001) for depressive symptoms and collectively associated with 62% and 53% of the mental health symptoms in men and women, respectively. Similar pattern was observed among pregnant women and 41% of the depressive symptoms were exclusively associated with those who reported household hunger. However, aORs associated with the concerns around pandemic and vaccine were mostly not significant and ranged from 1.12 to 1.26 which resulted substantially lower impacts on depressive symptoms (PAR%:7%-and-14%). Our findings suggest that South Africa still has unacceptably high rates of hunger which is accelerated during the pandemic. These results may have significant clinical and epidemiological implications and may also bring partial explanation for the low vaccine coverage in the country, as priorities and concerns are skewed towards economic concerns and food insecurity.

## Introduction

In addition to being home to more than seven million individuals living with Human Immunodeficiency virus (HIV), South Africa also has the highest burden of coronavirus disease 2019 (COVID-19) related comorbidities in Africa (Karim et al., 2009; Nwosu et al., 2022; Wand et al., 2018). Besides its devastating impact on physical health, the pandemic has also caused severe social and economic crisis worldwide (Broadbent et al., 2020; Wills et al., 2020). Increasing levels of employment, income and food insecurities have been widely reported, particularly in low- and- middle income countries (Ali et al., 2022; Gebeyehu et al., 2022; Mondal et al., 2021). Compared to the other parts of the world, African countries have the highest burden of food insecurities and reported hunger, which have increased substantially since the pandemic emerged (Chineka et al., 2021). South Africa has historically had severe economic and social problems (Aliber, 2009; Altman et al., 2009). One third of the population was estimated to be unemployed in 2020 January (Reuters, 2020).

As the burden of COVID-19 infections and related comorbidities continues its devastating impact globally, investigating the geospatial changes in socioeconomic conditions and their associations with adverse health outcomes, is crucial for understanding the ongoing impact of the pandemic. This has not been fully understood in South African context. The primary objective of the current study is to quantify the temporal changes in geographical variations in socioeconomic conditions including unemployment, food insecurity, and their combined impact on depressive symptoms among South Africans who participated into several rounds of national surveys. In our analysis, we also estimated the population-attributable risk percent ($PAR\%$) for various combinations of these risk factors after accounting for their correlation structure in multifactorial setting (Wand & Ramjee, 2012). In this context, PAR% can be interpreted as proportion of mental health symptoms that could be prevented if low socioeconomic conditions including food insecurity were improved (at least theoretically) in the target population. These analyses were also repeated in a cohort of pregnant women. Given the significant association between severe malnutrition and hunger and their impacts on adverse maternal health, the pandemic potentially have serious long-term implications which is yet to be assessed (Thurstans et al., 2022). Taken together, our study provides the most recent snapshot of the food insecurity, hunger, pre-existing comorbidities, concerns around the pandemic and vaccine and their relative contributions to mental health symptoms in South Africans.

## Literature review

South Africa has the highest burden of coronavirus disease 2019 (COVID-19) related comorbidities in Africa (Ngangue et al., 2022; Bradshaw et al., 2022). In addition to this, pandemic also had significant impact on food availability and access to food due to significant declines in unemployment rates and wages (Dunn et al., 2020). As a result poverty and food insecurity increased substantially worldwide including in South Africa (HRW, 2020). Following the first lock down on March 27, 2020, unemployment rate in South Africa increased to 41% which led higher levels of food insecurity and household hunger across the country (Hart et al., 2022). More than two and a half years into the pandemic, the related comorbidities during and post-acute infection continue to adversely impact physical health (Lake, 2020). There is also growing evidence for increasing levels of anxiety and depression worldwide which have been, in part, attributed to social and economic pressures caused by the pandemic (Bo et al., 2020; Camacho-Rivera et al., 2022; Fang et al., 2021). Strong association between poverty and anxiety/depression have been previously reported in other populations (Lund et al., 2010). In COVID-19 era, there is a growing evidence for increasing levels of mental health conditions where low-income families are particularly the most vulnerable populations (Nanama et al., 2012). South Africa also faced significant delays in vaccine allocation and delivery where vaccination programs officially started one year after vaccine approval, similar to the patterns observed in other low-and-middle-income countries (Rydland et al., 2022; Sen-Crowe et al., 2021). National estimates also revealed significant increase in excess mortality rates across the country which were primarily attributed to the direct or indirect impact of COVID-19 infections and related comorbidities across the country (samrc.ac.za).

## Study area

The study area was the country of the Republic of South Africa with all nine provinces: West Cape, North Cape, East Cape, North West, Free State, KwaZulu Natal, Mpumalanga, Gauteng and Limpopo.

## Data and methods

### Study populations

We used national surveys conducted among adult South Africans (18 years or older) since the pandemic emerged in 2020. The primary analysis used the data from the following surveys:

1. *The National Income Dynamics Study—Coronavirus Rapid Mobile Survey (NIDS-CRAM):* Five rounds of the NIDS-CRAM (N = 30,370) which were conducted during the period of May 2020–2021 (NIDS-CRAM 1–5).
2. *COVID-19 Vaccine Survey (CVACS) 1* (N = 3,510) and 2 (N = 3,608): two rounds of surveys conducted in South Africa in November 2021-February/March 2022 by the Southern Africa Labour and Development Research Unit (SALDRU). Details of the surveys and the populations were previously described (CVACS 1 and 2). A multistage stratified cluster sampling was used to select the households. Respondents were interviewed telephonically in all surveys.
3. *MomConnect mhealth platform:* Two rounds of cross-sectional national surveys conducted in June 2020 (N = 3,140) and July 2020 (N = 2,287) among pregnant women and new mothers who attended public antenatal care programs and included in MomConnect database (Sayed et al., 2022). Women participated to the surveys through the mobile “SMS” messaging and received ZAR10 for their time.

### Socioeconomic and health behaviours

Our analysis included: age (< 35, 35–49, 50–59 and 60 + years), ethnicity (Black vs. others) employment status, education, monthly household income, medical aid/insurance, smoking (yes/no). Participants’ COVID-19 risk-perceptions, beliefs and attitudes were measured by asking (1) *Do you think you will get very sick with covid-19 in the next 12 months?*; (2) “*I don’t trust vaccine/ Vaccine has side effects*”; (3) “*Do you intend to receive vaccine?*”; (4) “*May not be able to work if I get sick with vaccine?*”; (5) “*Did you run out of money to buy food in past two weeks*”?; (6) “*Anyone in house gone hungry because wasn't enough food*”? Self-perceived depressive symptoms were collected in Likert-scale using the Patient Health Questionnaire-2 (PHQ-2) assessment too (Kroenke et al., 2003). The PHQ-2 which was originated from the PHQ-9, has been used extensively and validated as an initial screening tool to identify individuals with depressive symptoms. The sensitivity of a cut point 2 or more had pooled sensitivity and specificity 91% and 70% respectively (Kroenke et al., 2003). Sensitivity and specificity of a cut point 3 ranged from 74 to 96% and 75% to 82% respectively Participants were asked “*Have you felt hopeless, down or depressed in the last seven days?*” and “Have you felt little interest or pleasure in doing things in the last seven days?” with the possible responses: “*Not at all*” (0-point), “*Several days*” (1-point), “*More than half the days*” (2-points), and “*Nearly every day*” (3-points). The current study used the cut point of ≥ 2 to classify the individuals as having depressive symptoms.

### Geographical data

Survey respondents resided in one of the nine provinces across South Africa. Study participants were geocoded (i.e. *latitude* and *longitude)* according to their residential areas, and districts, with no link to their names and addresses.

## Methods

### Descriptive and logistic regression models

We described and compared the characteristics of the study populations using the chi-square tests across the three time periods 2020, 2021 and 2022. Weighted logistic regression models were used to identify the significant correlates of “depressing symptoms” while accounting for the multistage sampling design. Adjusted odds ratios (aORs) from multivariable analysis and their 95% confidence intervals (CIs) were presented.

### Geoadditive models

Geospatial investigation of the data was conducted using the geoadditive models which fit a smoothed bivariate function of $latitude, longitude$ in logistic regression setting (Kammann & Wand, 2003; Wand et al., 2021, 2022). For a binary outcome: “unemployed” vs “employed” after adjusting for age, sex, and ethnicity:

$$\begin{aligned} & logit\left[ {P({\text{unemployed}}} \right)_{i} /\left( {1 - ~P\left( {{\text{unemployed}}} \right)_{i} } \right] = f(latitude_{i} ,~longitude_{i} ) \\ & \; + \beta _{1} \left( {age} \right)~ + ~\beta _{2} \left( {education} \right) + ~\beta _{3} \left( {ethinicity} \right) + \varepsilon _{i} \;\;\;i = 1, \ldots ,~n \\ \end{aligned}$$

for ith individual; in this setting $f$ is a smoothed bivariate function of latitude and longitude, ${\beta }_{1}{, \beta }_{2} \mathrm{and} {\beta }_{3}$ are regression coefficients for age, male and ethnicity; ${\varepsilon }_{i}$ error term $\sim Normal(0, {\sigma }^{2})$. Models produced predicted degrees of freedoms and *p*-values for $f$ which were referred as estimated $(e.d.f)$ where p < 0.05 considered as evidence for significant geographical variations. We used data-visualization techniques to present intensity of prevalence of an outcome of interest across the region (i.e. maps) which were created using the “Multivariate Generalized Cross Validation” (mgcv) (Wood et al., 2016).

### Population attributable risk percent (PAR%)

We estimated the population-level contributions of the most influential characteristics on depressive symptoms after accounting for the correlated nature of the risk factors in multifactorial setting where we combined the odds ratios from a standard logistic multivariable regression model and prevalence of an exposure(s) and estimated the population-attributable risk ($PAR\%$) (Wand et al. 2012):

$$PAR\% = \frac{p(OR - 1)}{{p(OR - 1) + 1}} = 1 - \frac{1}{{\sum\nolimits_{s = 1}^{2} {p_{s} OR_{s} } }}$$

where $OR_{s}$ is the odds ratio and $s$ corresponds to the two levels of a risk factor of interest. For a multi-level exposure:

$$PAR = \frac{{\sum\nolimits_{s = 1}^{S} {} p_{s} (OR_{s} - 1)}}{{1 + \sum\nolimits_{s = 1}^{S} {} p_{s} (OR_{s}^{{}} - 1) + 1}} = 1 - \frac{1}{{\sum\nolimits_{s = 1}^{S} {p_{s} OR_{s} } }}$$

## Results

Distribution of demographic and socioeconomic characteristics were described by three time points 2020, 2021 and 2022 (Table 1). The vast majority of the survey participants were Black South Africans (87% and 80% in 2020/2021 and 2022 respectively). The most recent survey participants were more likely to be unemployed and had lower education. Receiving COVID relief fund doubled in 2021 compared to 2020. Proportion of participants who reported lower household income (< ZAR3,500), lack of food in past 7 days and running out of money to buy food over the past month were also declined overtime (*p* < 0.001). Concerns around COVID-19 and safety of the vaccines increased from 26 to 39% (in 2020) and 31% to 58% (in 2022); while intention to receive vaccine declined from 34 to 18% (*p* < 0.001). Approximately one third of the study population in 2020 surveys rated their current health status moderately poor due to pre-existing chronic health conditions such as HIV, diabetes, asthma which declined to 25% in 2021. Prevalence of depressing symptoms appeared to increase overtime from 34 to 38% (*p* < 0.001). More than 70% of the survey population in 2022 did not have medical insurance or health coverage. In our prenatal cohort, 18% of the pregnant women reported household hunger in past seven days.

**Table 1:** Characteristics of the study population

	Surveys in 2020^¥^ *N* = 18,879	Surveys in 2021^¥¥^ *N* = 11,491	Survey in 2022^¥¥^ *N* = 3608	*p*-value
Sex				< 0.001
Male	39%	39%	53%	
Female	61%	61%	47%	
Ethnicity				< 0.001
Other^§^	13%	13%	20%	
Black	87%	87%	80%	
Age				< 0.001
< 30	28%	32%	20%	
30–39	29%	26%	33%	
40–49	21%	19%	16%	
50–59	10%	11%	17%	
60 +	12%	13%	13%	
Education level				< 0.001
Year 12 +	48%	47%	39%	
< 12 year	52%	53%	61%	
Employed/income past 30 days				< 0.001
Yes	39%	47%	44%	
No	61%	53%	56%	
Income last month				
ZAR 3500 +	–	33%	46%	< 0.001
< ZAR 3,500	–	67%	54%	
COVID relief fund received				< 0.001
No	79%	56%		
Yes	21%	44%	–	
Smoking regularly				
No	88%	–	–	
Yes	12%	–	–	–
Run out of money to buy food ^ξ^				< 0.001
No	48%	55%	60%	
Yes	51%	45%	39%	
Lack of food in past 7 days				< 0.001
No	74%	82%	81%	
Yes	26%	18%	19%	
Run out of money to buy food / lack of food past two weeks				< 0.001
No	45%	52%	56%	
Yes	55%	48%	44%	
I will get infected with COVID				< 0.001
No	74%	62%	61%	
Yes	26%	38%	39%	
Concerns for vaccines’ safety Vaccines				< 0.001
No	–	69%	42%	
Yes	–	31%	58%	
Intention to receive vaccine				< 0.001
No	–	66%	82%	
Yes	–	34%	18%	
Concerns for work related commitments due to the vaccine				–
No	–	–	50%	
Yes	–	–	50%	
Medical insurance/aid				
Yes	–	–	39%	
No	–	–	71%	
Mental health^§^				< 0.001
Anxiety symptoms	51%	60%	43%	
Depressing symptoms	34%	39%	38%	
Intention to receive vaccine				< 0.001
No	–	66%	82%	
Yes	–	34%	18%	
Current health condition ^ξ^				< 0.001
Good	68%	72%	75%	
Poor	32%	28%	25%	
May not be able to work if vaccinated				–
No	–	–	44%	–
Yes	–	–	56%	–

^¥^ CRAMS – May-December 2020^; ¥¥^ CRAMS – February-May 2021; ^¥¥¥^ CVACS 1 – February/March 2022; ^ξ^ Self-rated health status/having chronic conditions (e.g. HIV, diabetes, asthma); ^**§**^Measured using PHQ2; ^ξ^ past 30 days, which was measured 2020 and 2021 surveys only

### Temporal trends in geospatial variations in socioeconomic characteristics and concerns around

**Fig. 1:**
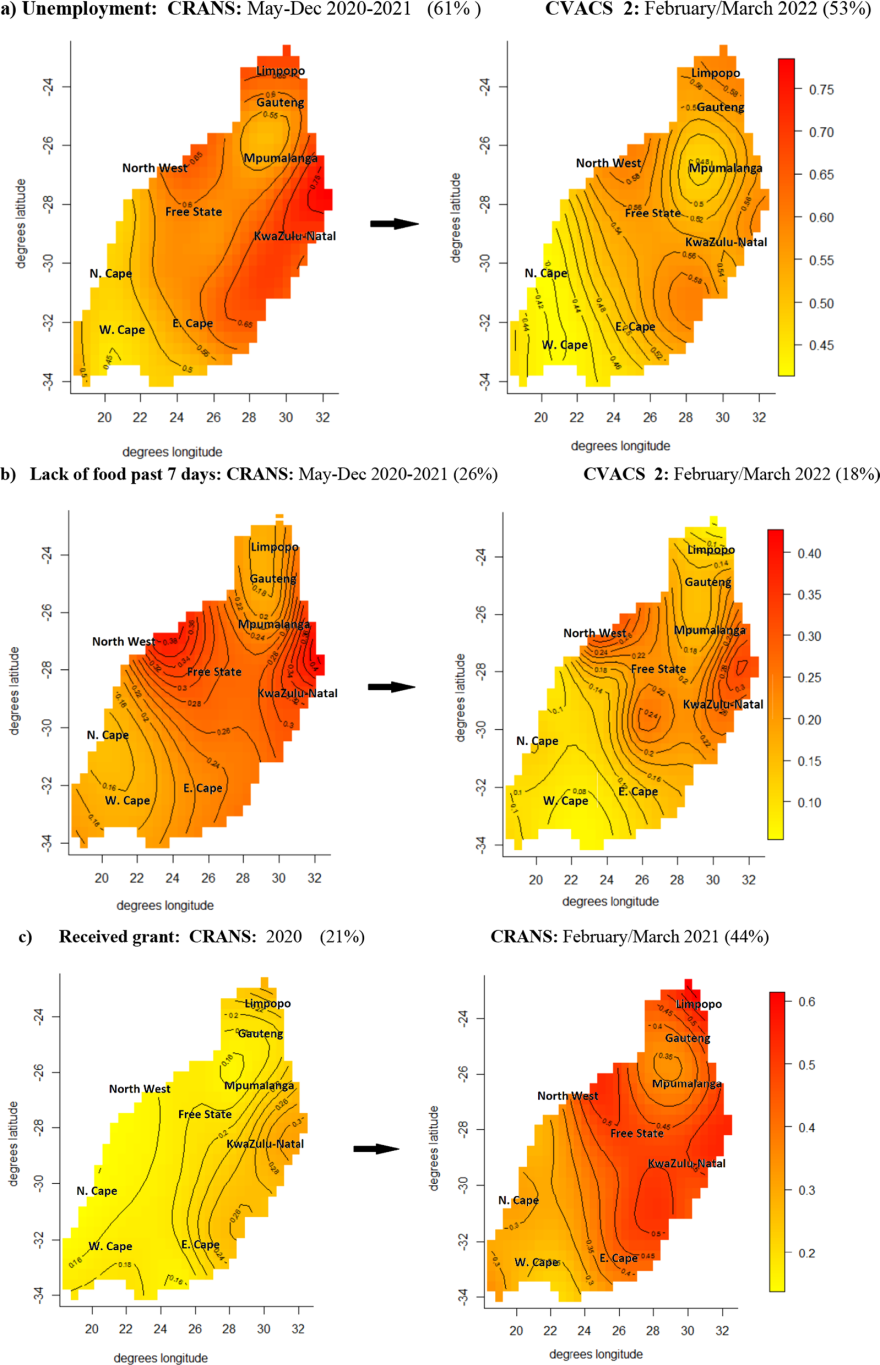
Temporal changes in geospatial variations in socioeconomic characteristics **a** Unemployment: CRANS: May-Dec 2020–2021 (61%) CVACS 2: February/March 2022 (53%) **b** Lack of food past 7 days: CRANS: May-Dec 2020–2021 (26%) CVACS 2: February/March 2022 (18%) **c** Received grant: CRANS: 2020 (21%) CRANS: February/March 2021 (44%)

Figure 1a–c presented the temporal changes in geospatial variations of the socioeconomic conditions including unemployment, lack of food and receiving COVID-19 relief fund. Overall 61% of the survey populations were unemployed in May–August 2020 with significant sub-geographical-level variations across the country ($e.d.f$. = 20.39, *p* < 0.001) (Fig. 1a, Table 2). Overall proportion of households with lack of food (past seven days) also reduced from 26 to 19% during the same period with significant geographical variation, which ranged from 20%-to-33% in 2020 and 16%-to-27% across the states ($e.d.f$. = 18.02 (2020) and $e.d.f$. = 20.21(2021), *p* < 0.001) (Fig. 1b, Table 2). Meanwhile, proportion of individuals who received COVID-19 relief funds increased from 26% in 2020 to 44% in 2022. Despite the statistically significant geographical variations, the maps revealed broadly uniform distribution of these characteristics with no apparent sub-district differences (Fig. 1c, Table 2).

**Table 2:** Estimated degrees of freedoms from generalized additive models

	“Unemployed”	
Surveys	Overall prevalence	Expected degrees of freedom (*p*-value)
CRANS: May–Dec 2020–2021	61%	20.39 (< 0.001)
CVACS 2: February/March 2022	53%	15.47 (< 0.001)
	“Hunger yes rates”	
CRANS: July–December 2020–2021	26%	18.02 (< 0.001)
CVACS 2: February/March 2022	18%	20.21 (< 0.001)
	“Received grant”	
CRANS: July–December 2020–2021	21%	15.15 (< 0.001)
CVACS 2: February/March 2022	44%	19.60 (< 0.001)

### Individual and population-level impacts of characteristics on depressive symptoms

Adjusted odds ratios for the depressive symptoms from the multivariable logistic regression models were presented in Table 3 (men) and Table 4 (women) respectively. There was an increasing trend in ORs across the younger age groups in all time points. Younger survey participants were significantly more likely to report depressive symptoms compared to those aged 60 years or older in both sexes. The odds ratios associated with ethnicity switched directions overtime where Black South Africans were significantly more likely to report mental stress in 2022 compared to the previous years. Being unemployed (aOR: 1.33 and 1.76 in 2021 and 2022 respectively), reporting lack of food in past 7 days (aORs: 2.44–3.10 for men and 2.06–2.50 for women) and running out of money to buy food in past two weeks (aOR:1.81 and 2.35 in 2020 and 2021) were all associated with increased odds of depressive symptoms. Survey participants without medical insurance (which was only asked in 2022 survey) were also more likely to experience depressive symptoms (aOR:2.36 and 1.74 for men and women respectively). In 2022, 62% and 53% of the depressive symptoms were collectively associated with the three socioeconomic indicators: (1) being unemployed, (2) lack of food and (3) not having medical insurance in men and women respectively. Reporting lack of food in past 7 days was identified as the most influential risk factor and exclusively associated with 41% of the depressive symptoms in pregnant cohort. However, the odds ratios associated with unemployment in this population was 1.20 (*p* = 0.197) with PAR%: 2% (0%, 4%). COVID-19 related concerns remained stable among women, where 20% of depressive symptoms were collectively associated with concerns around vaccines’ safety. Similar patterns were observed among the pregnant women where 26% of the depressive feelings were attributed to those who expressed concerns about COVID-19.

**Table 3:** Men: Correlates of depressive symptoms (feeling depressed, hopeless) Score > 2

	Surveys in 2020			Surveys in 2021			Survey in 2022		
	Adjusted OR^**¶**^(95% CI)	*p*-value	PAR%^§^ (95% CI)	Adjusted OR^**¶**^ (95% CI)	*p*-value	PAR% § (95% CI)	Adjusted OR^**¶**^ (95% CI)	*p*-value	PAR%^§^ (95% CI)
Age									
< 30	1.26 (1.02,1.57)	0.033	*	1.50 (1.10, 2.03)	0.009	*	1.47 (0.86, 2.50)	0.157	*
30–39	1.33 (1.07, 1.66)	0.010	*	1.54 (1.14, 2.10)	0.006	*	1.39 (0.87, 2.21)	0.170	*
40–49	1.34 (1.06, 1.70)	0.015	*	1.49 (1.07, 2.07)	0.018	*	1.56 (0.93, 2.63)	0.093	*
50–59	1.42 (1.01, 1.73)	0.043	*	1.35 (0.93, 1.97)	0.114	*	0.97 (0.56, 1.66)	0.899	*
60 +	1			1			1		
Ethnicity									
Black	0.45 (0.37, 0.54)	< 0.001	*	0.35 (0.27, 0.46)	< 0.001	*	2.42 (1.62, 3.62)	0.019	*
Others ^ρ^	1			1			1		
Employed/income past 30 days									
Yes	1			1					
No	1.01 (0.84, 1.22)	0.880	< 1%	1.33 (1.12,1.60)	0.002	13% (10%, 16%)	1.76 (1.31, 2.37)	< 0.001	27% (21%, 33%)
Lack of food in past 7 days									
No	1			1			1		
Yes	2.44 (2.10, 2.83)	< 0.001	22% (20%, 24%)	3.10 (2.47, 3.90)	< 0.001	26% (23%, 29%)	2.72 (1.87, 4.00)	< 0.001	24% (20%, 29%)
Lack of money to buy food past two-weeks							N/A		
No	1			1					
Yes	2.12 (1.87, 2.40)	< 0.001	31% (28%, 33%)	2.78 (2.31, 3.34)	< 0.001	38% (34%, 41%)			*
Medical insurance/aid	N/A			N/A					
Yes							1		
No							2.36 (1.68, 3.33)	< 0.001	48% (41%, 56%)
Combined impact of low socioeconomic conditions									
Unemployed/income + lack of money to buy food + Lack of food in past 7 days + lack of medical insurance	*	*	42% (38%, 45%)	*	*	56% (52%, 61%)	*	*	62% (56%, 67%)
COVID relief grant received							N/A		
Yes	1			1					
No	1.26 (1.04, 1.53)	0.020	16% (11%, 21%)	1.12 (0.94, 1.34)	0.191	8% (5%, 11%)			
I will get infected with COVID									
No	1			1			1		
Yes	1.43 (1.27, 1.62)	< 0.001	14% (12%, 16%)	1.58 (1.32, 1.89)	< 0.001	18% (15%, 22%)	1.15 (0.82, 1.61)	0.410	< 1%
Concerns for vaccines’ safety				N/A					
Yes	1						1		
No	1.49 (1.24, 1.78)	< 0.001	13% (11%, 16%)				1.12 (0.76, 1.65)	0.556	7% (3%, 17%)
Combined impact of COVID-19 and vaccine related concerns									
Won’t get infected with COVID + vaccines’ not safe	*	*	19% (16%, 22%)	*	*	21% (18%, 23%)	*	*	7% (2%, 18%)
Current health condition ^ξ^	N/A								
Good				1			1		
Poor				2.22 (1.83, 2.70)	< 0.001	25% (22%, 28%)	1.90 (1.53, 2.37)	< 0.001	20% (17%, 23%)
Smoking regularly									
No	1								
Yes	1.48 (1.22, 1.80)	< 0.001	11% (8%, 12%)	N/A			N/A		

^¶^Results from the multivariable logistic regression models; for the *p*-values > 0.05, odds ratios were adjusted for all the risk factors significantly associated with the symptoms of depressing feelings; ^ρ^ Whites, coloured, Indians; ^§^ Calculated for only modifiable risk factors; ^ξ^ Self-rated health status/having chronic conditions (e.g. HIV, diabetes, asthma); N/A: not available

**Table 4:** Women: Correlates of depressive symptoms (feeling depressed, hopeless) Score > 2

	Surveys^¥^: 2020			Surveys^¥¥^: 2021			Survey^¥^: 2022		
	Adjusted OR^¶^ (95% CI)	*p*-value	PAR%^§^	Adjusted OR^¶^ (95% CI)	*p*-value	PAR%^§^	Adjusted OR^¶^ (95% CI)	*p*-value	PAR%^§^
Age									
< 30	1.10 (0.93, 1.30)	0.270	*	1.10 (0.88, 1.38)	0.404	*	1.14 (0.63, 2.07)	0.656	*
30–39	1.32 (1.08, 1.59)	0.004	*	1.45 (1.17, 1.80)	0.001	*	1.29 (0.74, 2.24)	0.367	*
40–49	1.25 (1.05, 1.48)	0.011	*	1.32 (1.06, 1.65)	0.015	*	1.58 (0.84, 3.00)	0.158	*
50–59	1.52 (1.25, 1.84)	< 0.001	*	1.16 (0.90, 1.50)	0.248	*	1.16 (0.63, 2.13)	0.634	*
60 +	1			1					
Ethnicity									
Black	1			0.67 (0.55, 0.83)	< 0.001		1.62 (1.08, 2.41)	0.019	*
Others ^ρ^	1.52 (1.32, 1.75)	< 0.001	*	1		*	1		
Employed/income past 30 days									
Yes	1			1			1		
No	0.90 (0.78, 1.05)	0.174	< 1%	0.94 (0.81, 1.06)	0.281	*	1.69 (1.18, 2.41)	0.004	31% (23%, 41%)
Lack of food in past 7 days									
No	1			1			1		
Yes	2.06 (1.84, 2.31)	< 0.001	19% (17%, 20%)	2.38 (2.02, 2.81)	< 0.001	22% (20%, 25%)	2.50 (1.63, 3.82)	< 0.001	23% (18%, 28%)
Lack of money to buy food past two-weeks									
No	1			1			N/A		
Yes	1.81 (1.65, 2.00)	< 0.001	27% (24%, 29%)	2.35 (2.05. 2.70)	< 0.001	37% (33%, 39%)			
Medical insurance/aid	N/A			N/A					
Yes							1		
No							1.74 (1.18, 2.57)	0.005	36% (26%, 46%)
Combined impact of low socioeconomic conditions									
Unemployed/income + lack of money to buy food + Lack of food in past 7 days + lack of medical insurance	*	*	38% (33%, 43%)	*	*	48% (42%, 54%)	*	*	53% (44%, 62%)
COVID relief grant received							N/A		
Yes	1			1					
No	1.41 (1.23, 1.61)	< 0.001	17% (14%, 20%)	1.03 (0.90, 1.17)	0.692	1% (0%, 2%)			
I will get infected with COVID									
No	1			1					
Yes	1.58 (1.43,1.74)	< 0.001	18% (16%, 20%)	1.65 (1.44, 1.90)	< 0.001	20% (17%, 22%)	1.26 (1.02, 1.54)	0.029	7% (5%, 10%)
Concerns for vaccines’ safety									
No	1			N/A			1		
Yes	1.33 (1.15, 1.53)	< 0.001	9% (7%, 11%)				1.35 (0.64, 1.95)	0.101	11% (8%, 14%)
Combined impact of COVID-19 and vaccine related concerns									
Won’t get infected with COVID + vaccines’ not safe	*		21% (18%, 24%)	*	*	20% (16%, 23%)	*	*	14% (11%, 17%)
Current health condition ^ξ^	N/A								
Good				1			1		
Poor				2.17 (1.87,2.51)	< 0.001	26% (23%, 28%)	1.78 (1.23, 2.52)	0.001	17% (13%, 22%)
Smoking regularly									
No	1								
Yes	1.90 (1.32,2.72)	< 0.001	3% (2%, 4%)	–	–	–	–	–	–

^¶^Results from the multivariable logistic regression models; for the *p*-values > 0.05, odds ratios were adjusted for all the risk factors significantly associated with the symptoms of depressing feelings; ^ρ^ Whites, coloured, Indians; ^§^ Calculated for only modifiable risk factors; ^ξ^ Self-rated health status/having chronic conditions (e.g. HIV, diabetes, asthma); N/A: not available

## Discussion

Using the data from the nine rounds of national surveys (including two from the antenatal surveys), we quantified the temporal and geospatial changes in impact of the pandemic on socioeconomic conditions and mental health symptoms. Our findings provided a compelling evidence for high levels of food insecurity which was evident shortly after the first lockdown started on March 27, 2020. Approximately 60% of South Africans who participated in the 2020 surveys, reported food insecurity or household hunger, which declined to 44% in 2022 March. This decline was primarily attributed to the government pandemic relief grants which started in May 2020 with ZAR 300/child (US$16.35) and increased to ZAR 500/caregiver (US$27.26) in June (Nwosu et al., 2022; South African Government, 2021). As of February/March 2022, 19% of the survey population reported household hunger which was higher than the pre-pandemic levels of 14% with substantial geospatial disparities across the country (Nwosu et al., 2022). Particularly KwaZulu-Natal, the second most populous province in South Africa, had the highest burden of the pandemic where 33% of the population experienced household hunger just a month after the first lockdown. These estimates substantially exceeded that of the current global average of 8.9% (United Nations, 2020; World Bank, 2021). KwaZulu Natal is also known to be the epicenter of the HIV epidemic and other comorbidities including tuberculosis and cardiometabolic conditions (Wand et al., 2022; Karin et al., 2009). Based on the national estimates, the province ranked number one with respect to the excess mortality rates since the pandemic emerged in 2020 (South African Medical Research Council (samrc.ac.za); NDoH, 2022). As expected, areas with high levels of household hunger overlapped with the areas where high proportion of unemployment was reported. Our findings indicate that the impact of the pandemic in the areas with high background rates of HIV and low socioeconomic conditions accelerated a decline in already challenging health and socioeconomic conditions (Camacho-Rivera et al., 2022). In our study population, people with pre-existing health conditions including HIV, tuberculosis, and cardiometabolic conditions were approximately twice as likely to report depressive feelings. In fact, following the low socioeconomic conditions these characteristics were identified as the most influential risk factors on poor mental health conditions, where 17% to 26% of the depressive symptoms are attributed to those who had pre-existing comorbidities. These results are consistent with the previous research conducted in other populations where chronic health conditions were associated with developing mental health symptoms pre and post pandemic era (Camacho-Rivera et al., 2022; Remien et al., 2019). However, their population level impacts in the pandemic era was not assessed, as it was in this study.

Similar patterns observed in our prenatal cohort, women who reported household hunger (adult/children) in past seven days were five times more likely to experience depressive symptoms compared to those who did not have household hunger. In this population, 41% of the depressive symptoms were exclusively associated with household hunger. In sex-specific analysis, unemployment, food insecurity, household hunger and lack of medical insurance were identified as the most influential risk factors for depressive symptoms. These factors were collectively associated with 62% and 53% of the mental health symptoms in men and women, respectively. These findings indicated that men may have been affected by the lack of job and food insecurities more than the women who participated in the surveys. This modest difference may be partially explained by the fact that women received government funding at a significantly higher rate than men (34% vs. 50%, data not shown). Another plausible explanation is that male masculinity, which is often linked to “being a provider", may be a driver of sex-specific differences in mental health disorders (Cardoso et al., 2016; Hatcher et al., 2019). The association between mental health symptoms and low socioeconomic conditions including food insecurity and hunger has been reported in other populations (Lauren et al., 2021; Ling et al., 2022; Lund et al., 2010). For example, in a study conducted in June/July 2020 in the United States, low-income participants who had food insecurity were more than three times more likely to be at risk of depression and anxiety (Fang et al., 2021). Similar patterns were also observed prior to the pandemic where food insecurity has been linked to mental and physical disorders (Ramezani et al., 2017)**.** In a study based on an econometric analysis, hunger correlated with “worsening health” in a cohort of South Africans **(**Nwosu et al., 2022**)**. However, our study is the first study to quantify the population level impacts of food insecurity on mental health symptoms that has been directly correlated to the pandemic.

One of the most important findings from our study is the weak associations between the depressive symptoms and the concerns around the pandemic and vaccine safety. In men, 19% of the mental health conditions were collectively attributed to the concerns around the pandemic and vaccine safety which declined substantially overtime to 7%. Similar patterns were observed among women (21% and 14%). These estimates are substantially lower than the combined impacts of food insecurity and hunger on increasing depressive feelings. The same was true in our perinatal cohort. Our findings may be interpreted as the survey participants continue to have higher concerns about food insecurity rather than the pandemic itself and safety of the vaccine. These results may have significant clinical and epidemiological implications and may bring partial explanation for the low vaccine coverage in the country, as priorities and concerns are skewed towards economic concerns and food insecurity. As of September 2022, less than half of the South Africans have received at least one dose of the vaccine which is substantially lower than the global targets of 70%. In our survey populations, intention to receive vaccine declined from 34% in 2021 to 18% in 2022. In addition, half of the 2022 survey participants reported that they do not intend to get vaccinated because of the work-related commitments which may also be linked to the fear for unemployment and food insecurity rather than concerns around vaccine safety. Although some of these findings are consistent with previous research, most of them are unique to the current study.

## Limitations

Our study has several limitations. First, the vast majority (> 85%) of the survey populations identified themselves as Black, therefore the other ethnicities were underrepresented. Some of the characteristics were not collected in all survey populations. All the characteristics, beliefs and attitudes were self-reported including the mental health symptoms, therefore they are subject to recall bias. Depressive symptoms were assessed using the PHQ-2 rather than more detailed PHQ-9. We used the multifactorial version of $PAR\%,$ which can account for the correlated nature of the risk factors considered in a multivariable model. Although we were able to compare some of these results with previous research, most of our findings are novel to this study. Therefore, direct comparisons were not possible.

Our study provided compelling evidence for immediate and severe effect of the pandemic where 27% of participating South Africans reported household hunger shortly after the pandemic emerged in 2020 which is substantially higher than the global average of 8.9%. As of March 2022, the country is substantially behind the “Sustainable Development Goals” set by the world leaders which aimed to “*end hunger, achieve food security and improved nutrition and promote sustainable agriculture*” by 2030 (United Nations, 2020).

Although we estimated the immediate and ongoing impact of the pandemic, our findings may potentially have important implications for its long-term impact which is currently not known. There is extensive literature that link hunger to severe malnutrition which is known to be the leading cause of growth failure including wasting, stunting, and other physical disabilities (Benjamin‐Chung et al., 2020; Bergeron et al., 2012; Bhutta et al., 2016). In our perinatal survey population, 18% of the pregnant women reported household hunger (adults/children) which was as highest in Eastern and Northern Capes, 24% and 27%, respectively. Given the significant impact of severe malnutrition on adverse maternal health, our findings may have important clinical and public health implications as the pandemic continues on. regarding the for the long-term impact of the pandemic (Benjamin‐Chung et al., 2020; Bergeron et al., 2012). These findings may indicate the potential increases in childhood growth failures which will have long term and wide side impact on the social, economic and health of the general population.

## Conclusion & implications

After decades of efforts to reduce the hunger globally, South Africa still has unacceptably high rates of hunger which is accelerated during the pandemic. The fear of food insecurity and hunger outweighed the fear of the pandemic among South Africans and may have impacted health behaviours including the getting vaccinated. As the global targets of get to ZERO Hunger is 8 years away, high rates of food insecurity and hunger appeared to be one of the greatest challenge that South Africans face, particularly in the pandemic era. Adding to this, is the sustained mental health impacts due to the pandemic, with higher rates of depressive symptoms among those who are most impacted by food insecurity, hunger and unemployment. Investigating the impact of the pandemic on socioeconomic status, and mental health and their population-level contributions in a nationally representative sample might have significant implications for planning, and modifying the current and future socioeconomic support programs, mental health programs and hunger prevention programs by targeting those at greatest risk of adverse outcomes.

## Data Availability

Publicly available data can be requested and accessed via https://www.datafirst.uct.ac.za/

## Declarations

### Human participation and/or animals

Not applicable. Unidentifiable publicly available data were used.
